# Insertions or Deletions (Indels) in the *rrn* 16S-23S rRNA Gene Internal Transcribed Spacer Region (ITS) Compromise the Typing and Identification of Strains within the *Acinetobacter calcoaceticus-baumannii* (Acb) Complex and Closely Related Members

**DOI:** 10.1371/journal.pone.0105390

**Published:** 2014-08-20

**Authors:** Christopher Maslunka, Bianca Gifford, Joseph Tucci, Volker Gürtler, Robert J. Seviour

**Affiliations:** 1 Biotechnology Research Centre, La Trobe University, Bendigo, Victoria, Australia; 2 School of Applied Sciences, Bundoora Campus RMIT University, Bundoora, Australia; St. Petersburg Pasteur Institute, Russian Federation

## Abstract

To determine whether ITS sequences in the *rrn* operon are suitable for identifying individual *Acinetobacter* Acb complex members, we analysed length and sequence differences between multiple ITS copies within the genomes of individual strains. Length differences in ITS reported previously between *A. nosocomialis* BCRC15417^T^ (615 bp) and other strains (607 bp) can be explained by presence of an insertion (indel 13i/1) in the longer ITS variant. The same Indel 13i/1 was also found in ITS sequences of ten strains of *A. calcoaceticus,* all 639 bp long, and the 628 bp ITS of *Acinetobacter* strain BENAB127. Four additional indels (13i/2–13i/5) were detected in *Acinetobacter* strain c/t13TU 10090 ITS length variants (608, 609, 620, 621 and 630 bp). These ITS variants appear to have resulted from horizontal gene transfer involving other *Acinetobacter* species or in some cases unrelated bacteria. Although some ITS copies in strain c/t13TU 10090 are of the same length (620 bp) as those in *Acinetobacter* strains b/n1&3, *A. pittii* (10 strains), *A. calcoaceticus* and *A. oleivorans* (not currently acknowledged as an Acb member), their individual ITS sequences differ. Thus ITS length by itself can not by itself be used to identify Acb complex strains. A shared indel in ITS copies in two separate *Acinetobacter* species compromises the specificity of ITS targeted probes, as shown with the Aun-3 probe designed to target the ITS in *A. pitti.* The presence of indel 13i/5 in the ITS of *Acinetobacter* strain c/t13TU means it too responded positively to this probe. Thus, neither ITS sequencing nor the currently available ITS targeted probes can distinguish reliably between Acb member species.

## Introduction

*Acinetobacter* spp. are important nosocomial pathogens and epidemiological survey data from around the world suggest that multi-antibiotic resistant *Acinetobacter baumannii* (genomic species 2), *A. pittii* (genomic species 3) and *A. nosocomialis* (genomic species TU13) are the most important clinically [1], [2], [3]. These three species were grouped with *A. calcoaceticus* (genomic species BG1), a soil organism, into the *A. calcoaceticus-A. baumannii* (Acb) complex because of their close genetic similarities [4], [2]. Based on DNA-DNA hybridization and ribotyping data, Gerner-Smidt and Tjernberg [5] proposed that two additional genomic species be added to the Acb complex, namely ‘close to 13TU’ and ‘between 1 & 3’. The taxonomic relationships of these strains to the other members of the Acb complex still have to be resolved [6].

Interest in using chemotaxonomic and molecular methods to distinguish between Acb complex members has increased, as phenotypic characters have generally proved to be unreliable for this task. Many such methods have been described [7], and include exploiting the sequence variations in the 16S–23S rRNA gene internal transcribed spacer region (ITS sequences) in the *rrn* operons [8]. In their analyses of *Acinetobacter* ITS regions, Chang et al. [9] suggested those of individual Acb member species were highly conserved in both their lengths and sequences. As intra-genomic ITS sequence variance had never been confirmed then in member species of this group, they proposed that the direct sequencing of Acb ITS sequences provided a promising method for their identification [9]. Although all *Acinetobacter* spp. are known to possess multiple ITS copies [10], [11], ITS targeted probes have been designed to identify Acb members [12]–[17] on the premise that the extent of Acb intra-species ITS copy sequence variance is low.

Methods based on ITS features include using multiplex PCR [13], oligonucleotide arrays [14], [15], oligonucleotide hybridisation probes [16], and microsphere-based arrays [17]. While nine of 12 currently available Acb probes have been claimed to give unequivocal identifications, the remaining three published ITS-targeted probes are more problematic. The *A. nosocomialis-*targeted P-13 probe [17] cross hybridized with the ITS sequences of some *A. pittii* strains, while the *A. pittii*-targeted Aci3 [16] and P-3 [17] probes cross hybridized with the ITS sequences of some *A. nosocomialis* strains. An examination of these three probe sequences against the ITSs of the type strain of *A. nosocomialis* (BCRC 15417^T^) and *A. pittii* (BCRC 15420^T^) revealed such cross hybridisation events should not have occurred.

The known ITS lengths of most Acb members appear to be highly conserved with *A. calcoaceticus* (6 strains) all 638 bp; *A. baumannii* (27 strains) all 607 bp; *A. pittii* (6 strains) all 619 bp [9]. However, while the majority of the publicly available ITS sequences of the *A. nosocomialis* strains are 607 bp, those of the type strain *A. nosocomialis* BCRC15417^T^ are 615 bp (Table 1). Furthermore, the differences in sequence between the 607 bp and 615 bp *A. nosocomialis* ITSs must be substantial as such a small (8 bp) length difference between otherwise identical ITS >600 bp would give a similarity score of >98%, and not the reported 95% [18]. Whether this variance is the result of point mutations or recombination events has never been examined.

**Table 1:** ITS of *A. calcoaceticus*, *A. baumannii*, *A. pittii* and *A. nosocomialis* strains downloaded from GenBank and their ITS length (bp).

		Acinetobacterstrain	AccessionNumber	ITSLength	Author
**A. nosocomialis**	(1)	00574	AY510070	607 bp	Chang et al, 2005 [9]
		v104-2	AY510071	607 bp	Chang et al, 2005 [9]
		DR25612/96	EU030649	607 bp	Koh et al, unpublished
		DR32226/96	EU030656	599 bp(2)	Koh et al, unpublished
		DB30014/96	EU030653	599 bp(2)	Koh et al, unpublished
		DM18619/96	EU030654	572 bp(2)	Koh et al, unpublished
		74510	FJ360743	607 bp	Zarrilli et al, 2009 [18]
		Ab22222^T(1)^	AKAR00000000	608 bp	Murphy et al, unpublished
	(2)	BCRC15417^T^	AY601830	615 bp	Chang et al, 2005 [9]
		BCRC15417^T^	U60281	609 bp(2)	Lagatolla et al, 1998 [16]
		NCTC 8102(1)	AIEJ00000000	615 bp	Chan et al, 2012 [30]
		Ab22222(1)	AKAR00000000	616 bp	Murphy et al, unpublished
		TG21145(1)	AMJH00000000	615 bp	Sahl et al, unpublished
**A. calcoaceticus**	(3)	ATCC 23055	U60278	628 bp(2)	Lagatolla et al, 1998 [16]
		LMG 1046	AY601820	638 bp	Chang et al, 2005 [9]
		LMG 992	AY601821	638 bp	Chang et al, 2005 [9]
		BCRC 11562	AY601822	637 bp	Chang et al, 2005 [9]
		IH9	FJ786266	629 bp(2)	Peix et al, 2009 [24]
		OCI1	FJ786267	632 bp(2)	Peix et al, 2009 [24]
		PHEA-2(1)	NC_016603	620 bp	Zhan et al, 2011 [44]
**A. baumannii**	(4)	BCRC10591	AY601823	607 bp	Chang et al, 2005 [9]
		BCRC15884	AY601824	607 bp	Chang et al, 2005 [9]
		BCRC15886	AY601825	607 bp	Chang et al, 2005 [9]
		LMG984	AY601826	607 bp	Chang et al, 2005 [9]
		ATCC 17978(1)	CP000521	608 bp	Smith et al, 2007 [45]
		ACICU(1)	CP000863	608 bp	Iacono et al, 2008 [46]
		AYE(1)	CU459141	608 bp	Vallenet et al, 2008 [47]
		SDF(1)	CU468230	608 bp	Vallenet et al, 2008 [47]
		BCRC10591	U60279	610 bp	Lagatolla et al, 1998 [16]
		DM19034/01	EU030648	607 bp(2)	Koh et al, unpublished
		DU32993/01	EU030651	607 bp(2)	Koh et al, unpublished
		DM169/96	EU030652	601 bp(2)	Koh et al, unpublished
		DU54004/05	EU030657	607 bp	Koh et al, unpublished
		DM19305/01	EU030660	602 bp(2)	Koh et al, unpublished
		DU5377/05	EU030658	601 bp(2)	Koh et al, unpublished
		DB6474/05	EU030659	607 bp	Koh et al, unpublished
		DU16891/96	EU030661	607 bp	Koh et al, unpublished
		DB15354/07	EU030662	601 bp(2)	Koh et al, unpublished
		DB34441/07	EU030663	600 bp(2)	Koh et al, unpublished
		25001 CMCC(B)	DQ108593	608 bp	Chen et al, 2007 [13]
		29108 CMCC(B)	DQ108594	608 bp	Chen et al, 2007 [13]
**A. pittii**	(5)	LMG1035^T^	AY601827	619 bp	Chang et al, 2005 [9]
		CCUG26384	AY601828	619 bp	Chang et al, 2005 [9]
		BCRC15420	AY601829	619 bp	Chang et al, 2005 [9]
		BCRC15420	U60280	622 bp	Lagatolla et al, 1998 [16]
		DB60079/01	EU030647	615 bp(2)	Koh et al, unpublished
		DM22501/01	EU030650	595 bp(2)	Koh et al, unpublished
		DM21785/01	EU030655	619 bp	Koh et al, unpublished
		SH024	NZ_GG753607	571 bp(2)	Peleg et al, 2012 [48]
		DSM 9306(1)	AIEF00000000	620 bp	Chan et al, 2012 [30]
		D499(1)	AGFH00000000	620 bp	Chen et al, 2012 [49]
		DSM 21653(1)	AIEK00000000	620 bp	Chan et al, 2012 [30]
		TG6411(1)	AMJI00000000	620 bp	Sahl et al, unpublished
**A. oleivorans**		DR1(1)	NC_014259	620 bp	Jung et al, 2010 [50]

(1) Whole genome sequences (from which all ITS were extracted *in* *silico*).

(2) Partial sequence.

(3) ITS sequences downloaded from GenBank were analysed using Geneious Pro (percentage similarity calculations not shown; accession numbers listed).

No instances of ITS allele sequence variance have been demonstrated for any member of this Acb complex. However, the unusual nature of the *A. nosocomialis* BCRC15417^T^ ITS, together with the low specificities of some published Acb ITS targeted probes suggested that a more thorough examination of the ITS of Acb members’ was required. The outcomes are reported here.

## Materials and Methods

### Strains sequenced and source of ITS sequences

The ITS sequences were analysed from the following strain collections: Collection (A): consisted of strains whose ITS sequences were downloaded as individual sequences, or extracted from whole genome sequence data, with the GenBank accession numbers shown in Table 1. The ITS of type strains sequenced by Chang et al. [9] were designated by them as *A. calcoaceticus* LMG1046^T^, *A. baumannii* BCRC10591^T^ and *A. pittii* LMG10350^T^, while the same strains sequenced here are designated as *A. calcoaceticus* ATCC23055^T^, *A. baumannii* ATCC19606^T^ and *A. pittii* ATCC19004^T^ (Table 1).

Collection (B): included were *Acinetobacter* strains in culture collections from which cloned ITS PCR amplicons were generated and sequenced here. They contained strains from three *Acinetobacter* species and two genomic species included in or closely related to the Acb complex. These were *A. calcoaceticus* strains ATCC23055^T^, 97366, 97420, 97424; *A. baumannii* strains ATCC19606^T^, 97429, 97434, 16842; *A. pittii* ATCC19004^T^; ‘close to 13TU’ strains 5804 and 10090 (hereafter referred to as c/t13TU 5804 and c/t13TU 10090); ‘between1&3’ strains 10095 and 10169 (hereafter referred to as b/n1&3 10095 and b/n1&3 10169); *Acinetobacter* BENAB127. Thirteen of these Acb strains were speciated by DNA-DNA hybridization [19], [20], [5], and 11 were isolated from clinical samples, as detailed by Maslunka et al. [10]. The exceptions were *A. calcoaceticus* strains 97366 and 97420, both isolated from soil, and *Acinetobacter* strain BENAB127, which was isolated from activated sludge. While Beacham et al. [21] identified BEN127 phenotypically as *A. pittii*, analysis of its 16S rRNA (data not shown: Accession number HE651911) and ITS sequences (see later) suggest it cannot be placed confidently in this species. Consequently, it is referred to here as *Acinetobacter* BENAB127.

Collection (C): These forty-one clinical isolates were ‘identified’ tentatively by pulsed field gel electrophoresis (PFGE) and riboprinting as either *A. baumannii* (7–8, 147–150, 153–8, 160–1, 163–8, 170–1, 176–8, 180–1, 248–9, 251, 253–7, 262) or *A. calcoaceticus* (250, 175, 151, 159, 169) at the Austin Hospital Microbiology Department, Melbourne, Australia. 16S rRNA sequencing performed in this study (data not shown) revealed that only five strains (7, 8, 147, 148, & 150) were considered to belong to *A. baumannii*. Strains 151, 159, 169, and 175 and 250 did not appear to be members of either of these species. Instead, 16S rRNA sequences of strains 151, 159 and 175 were most similar to those of *A. pittii* and *A. oleivorans*, while those of strains 169 and 250 clustered most closely to *Acinetobacter* b/n1&3 strain 10095 and *A. calcoaceticus* respectively (data not shown). The remaining 31 were considered most likely to belong to *A. nosocomialis,* a conclusion supported by ITS sequencing data (see later). The ITS sequences of all these were generated by direct sequencing as described next (accession numbers given below).

### DNA extraction

Cells of each collection (B) strains were grown on R2A agar at 30°C. Genomic DNA was extracted using an UltraClean Soil DNA extraction kit (Mo Bio Laboratories Inc.) and stored in 10 mM Tris buffer at −20°C.

### Direct sequencing of the ITS

ITS sequences were PCR amplified by adding genomic DNA to a reaction mixture containing the 1512f (GTC GTA ACA AGG TAG CCG TA) and 6r (GGG TTY CCC CRT TCR GAA AT) bacterial universal primers [9]. This protocol generated ITS amplicons containing 16S and 23S rDNA flanks of approximately 18 bp and 115 bp respectively. A GeneAmp PCR 2400 thermocycler (Applied Biosystems) was used, with initial denaturation at 94°C for 6 min, denaturation at 94°C for 30 sec, annealing at 53°C for 1 min, extension at 72°C for 30 cycles of 90 sec each and a final extension at 72°C for 10 min. Amplicons were cleaned using an Ultra-Clean PCR Cleanup Kit (Mo Bio Laboratories Inc.).

Product purity was checked by electrophoresis with 2% agarose gels stained with ethidium bromide, which were viewed with a UV transilluminator. DNA concentrations were calculated using Adobe Photoshop 7 to compare the band intensity of each ITS amplicon against that of similarly sized 2 Log ladder band (New England Biolabs) of known DNA concentration. Primer 1512f was used for forward reads while primer 6r was used for reverse reads. Samples were sequenced by the Australian Genome Research Facility (AGRF) (Brisbane, Australia).

### ITS sequencing by colony PCR

Ligation (pGem-T Easy Vector kit) and transformation were carried out as described by Carr et al. [22]. Cells from individual colonies were transferred from the growth medium (LB plates with ampicillin/IPTG/X-gal) and resuspended in 10 mM Tris buffer. Cells were collected from clones for each strain, and shaken gently for 60 min before being stored at 4°C. The M13f (CGC CAG GGT TTT CCC AGT CAC GAC) and M13r (TCA CAC AGG AAA CAG CTA TGA C) primers were used to amplify the ITS sequence as above, but with denaturation occurring at 95°C for 30 sec. PCR products were cleaned, checked for purity on 2% agarose gels and prepared for sequencing as described earlier.

### ITS sequence accession numbers

All sequences generated in this study were deposited in the DDBJ/EMBL/GenBank database (at EMBL-EBI, European Molecular Biology Laboratories, http://www.ebi.ac.uk/embl/) as follows: Strain collection (B). *A. calcoaceticus* strains, HE651573–HE651628 and HE651903–HE651906; *A. baumannii* strains, HE651629–HE651686 and HE651907–HE651910; *A. pittii* ATCC19004^T^, HE651687–HE651696 and HE651911; c/t13TU 5804 and c/t13TU 10090, FN669512–FN669527 and FN677952–FN677963; b/n1&3 10095 and b/n1&3 10169, HE651546–HE651572, HE651912 and HE651913; *Acinetobacter* BENAB127, HE651697–HE651710 and HE651914. Strain collection (C) strains 41 directly sequenced clinical isolates; 7–8, KC257006–7; 147–151, KC257008–12; 153–161, KC257013–21; 163–171, KC257022–30; 175–178, KC257031–34; 180–181, KC257035–36; 248–251, KC257037–40; 253–257, KC257041–45; 262, KC257046.

### In silico analysis of ITS sequences

Geneious Pro (version 4.0.4: www.geneious.com) bioinformatics software (Biomatters Ltd) was used to construct ITS sequence alignments, contigs and consensus sequences. Similarity matrices were constructed with MegAlign (DNASTAR software, version 7.1) using a ClustalW alignment. Only a single ITS sequence was publicly available for most *Acinetobacter* species, including those with variable length ITS sequences [9].

### Assessing *A. pittii* ITS targeted Aun3 probe specificities

ITS amplicon generation was performed with the same PCR protocol as described above, except that a 70 sec extension time was applied, and probe Aun3 [14] (GAT GAA GAA TCG CAC GGA CAA CA) as reverse primer. PCR products were separated on a 2% agarose gel stained with ethidium bromide.

## Results

### ITS sequence isolation and analysis

**Figure 1:**
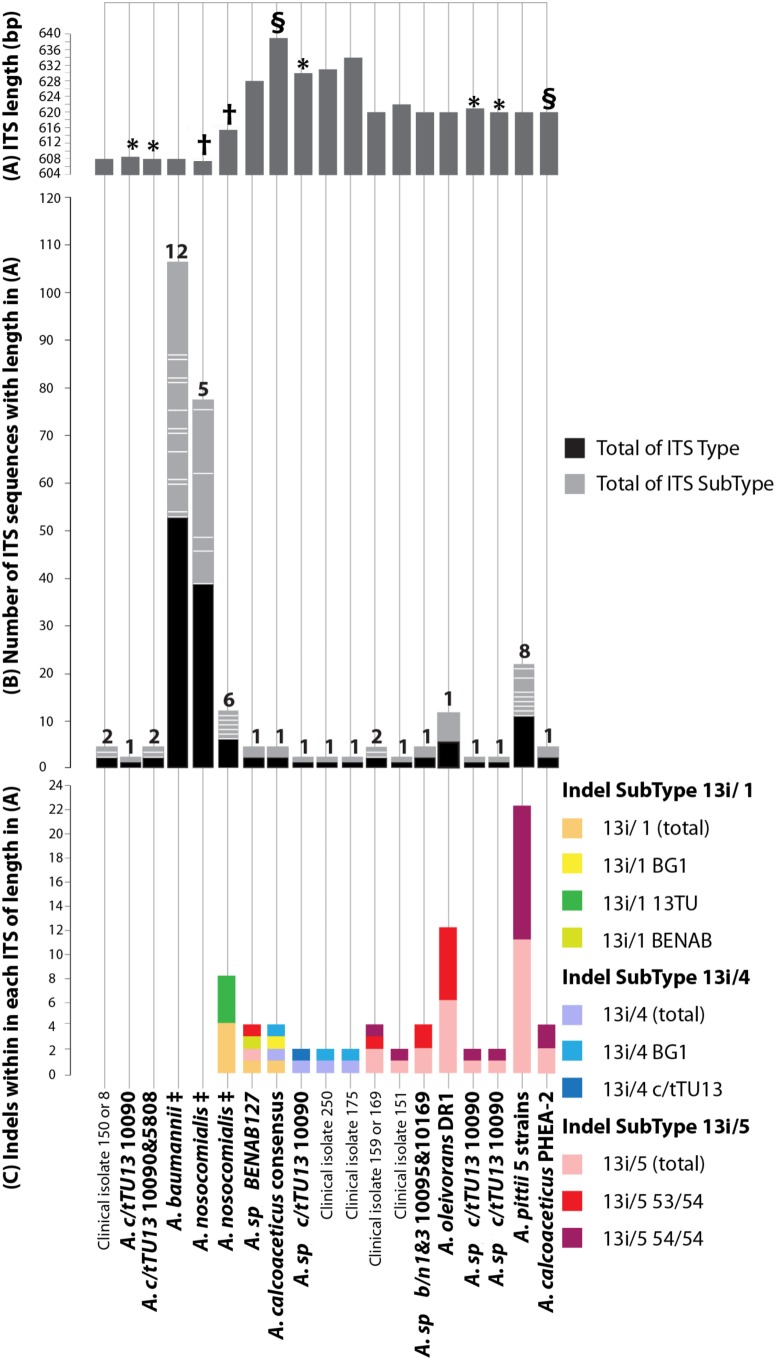
Sequence properties of ITS *Acinetobacter* Acb member strain sequences with one or more distinctive properties. ITS-Type, ITS-Subtype, Indel Subtype and Indel Sequence Subtype defined in Results. ITS sequences were plotted with species on y-axis with established species in bold and collection C isolates in regular type. Figure 1A shows their individual ITS sequence lengths, Figure 1B the number of ITS sequences of a distinctive length (black) or sequence (grey) and Fig. 1C the number of indels (see Results) within each ITS of a distinctive length. The Acb species and strains associated with each ITS sequence length, number, sequence and indel type are given below. On x-axis: (A) ITS-lengths (named ‘Type’ and symbols refer to: * A. c/tTU13; § A. *calcoaceticus*; † A. *nosocomialis*); (B) Number of ITS-Types and -SubTypes (ITS copies of same sequence lengths but different sequences). Black/grey bars (parallel to green bar in A) show 40 ITS Types and 5 SubTypes of *A. nosocomialis* 607 bp ITS; black and grey bars (parallel to purple bar in A) show 5 ITS Types and 5 SubTypes of *A. nosocomialis* 615 bp ITS; white lines show number of each SubType; (C) Number of specific Indel Subtypes and Sequence Subtypes as totals for each strain are shown by coloured bars. See Fig. 3 for sequences; each indel subtype name refers to strains with 13i/1 and 13i/4 indels. 13i/5 53/54 refers to homology in 53/54 nucleotides, while 13i/5 54/54 indicates sequence homology for all 54 nucleotides). ‡ Refers to *A. baumannii* strains in Table 1 and M&M, isolates 147–8, 7; *A. nosocomialis* 607/8 bp, 00574, v104-2, DR25612/96, DR3226/96, DM18619/96, 74510, AB22222, isolates 149, 153–6, 160, 163–8, 170–1, 176–8, 180–1, 248–9, 251–7; *A. nosocomialis* 615/6 bp, TG21145, BCRC15417^T^, AB22222, NCTC8102, isolate 157; *A. pittii*, DSM9306, D499, DSM21653, TG6411, SH024. * Property defined as: ‘Species’, ‘Strain’, ‘Type’, ‘SubType’, ‘Indel’, ‘Indel sequence subtype’, ‘rrn allele name’. ¶ With ITS sequence Types of different lengths (608 to 638 bp), depending on their length and/or sequence, between 90–100% of the sequence is homologous, allowing for precise alignment. The alignment program introduces indels into regions not homologous with high numbers of other sequences in these regions, depending on ITS sequence Type length. Details in Gürtler & Grando [42] and Gürtler et al [43].

Organizational properties of 139 Acb ITS sequences with one or more distinguishing features are shown in Figure 1. When genomic DNA was used as PCR template for the *Acinetobacter* strains in strain collections (B) and (C), electrophoretic mobilities for ITS amplicons (approximately 800 bp long, data not shown) from *A. calcoaceticus* ATCC23055^T^, *A. baumannii* ATCC19606^T^ and *A. pittii* ATCC19004^T^ matched exactly those reported for these same strains by Chang et al. [9]. Furthermore, with total genomic DNA as PCR template, direct ITS sequencing of strains in collection (B) was successful for every strain except c/t13TU 10090 (discussed below), and their ITS sequences derived by this approach were identical to those derived from sequencing multiple individual clones of the same strains (see below).

**Figure 2:**
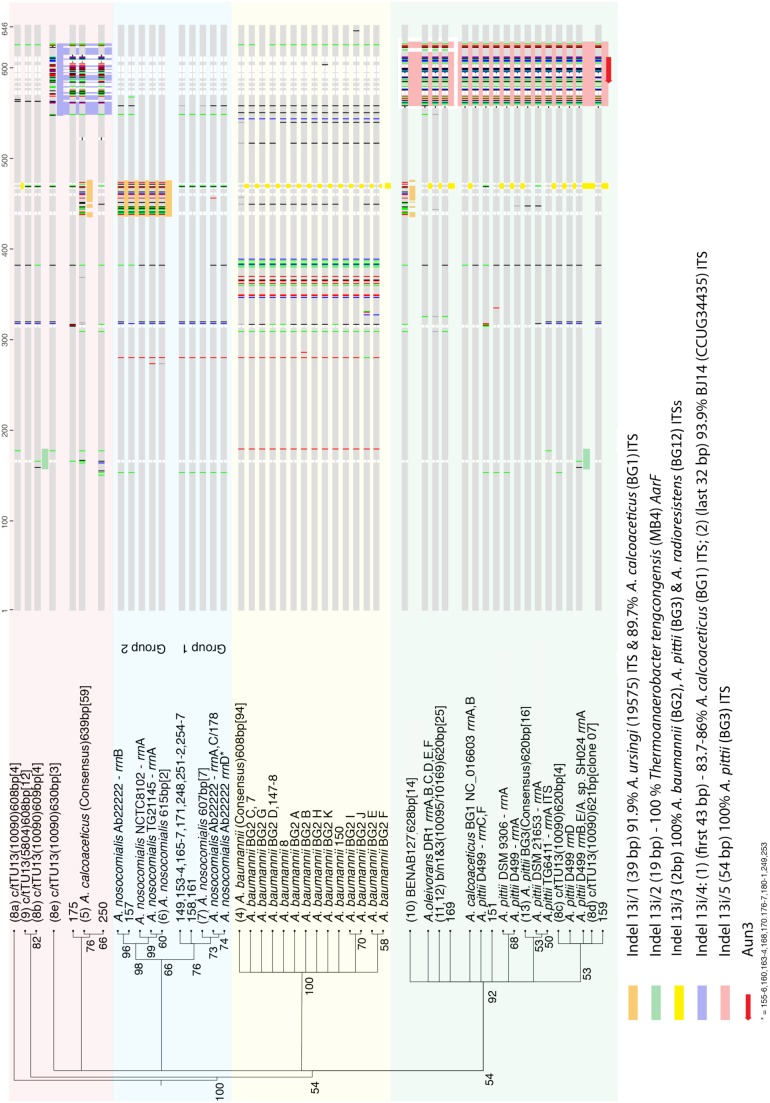
Sequence alignments of distinctive ITS subtypes from ITS sequences of Acb members (see Figure 1). Indels labeled as in Figure 1. Nucleotide substitutions [grey bars, homologous regions; vertical lines, red (T), green (A), black (G), blue (C), grey (N)]. For each indel, the closest Blast match is listed below its name. Sequences given only numbers are those of the collection C clinical isolates. The number of ITS sequences used in each consensus sequence is given in parentheses [ ]. The phylogenetic tree was constructed by Neighbor-Joining with 100 boot straps and numbers at nodes show percentage confidence of respective branches. Branch lengths are proportional to number of nucleotide substitutions but make no allowance for deletions (shown in white on the alignment).

After 16S and 23S rRNA gene flanks were removed, the resultant ITS consensus sequence lengths were as follows: 638 bp/639 bp, for all sequenced *A. calcoaceticus* strains; 608 bp, for all sequenced *A. baumannii* strains; 620 bp, for *A*. *pittii* ATCC19004^T^; 608 bp, for strain c/t13TU 5804; 620 bp, for both ‘b/n1&3’ strains; and 628 bp, for *Acinetobacter* BENAB127. All these ITS consensus sequences contained a tRNA^ile^ gene (position 61/62–137/138) and tRNA-^ala^ gene (position 193/194–268/269) as shown in Figure 2.

Direct sequencing of the ITS sequence of strain c/t13TU 10090 generated sequence data that suggested amplicon contamination, where read quality deteriorated markedly downstream of the first 268 bp. As with the strain c/t13TU 5804 ITS sequence, tRNA^ile^ and tRNA^ala^ genes were located at positions 61 to 137 and 193 to 268 respectively. Subsequent cloning and sequencing revealed these initial results were not the result of contamination, but rather by the presence of multiple variable-length ITS sequences within the c/t13TU 10090 genome (discussed below).

Clone sequencing was performed on PCR products amplified from genomic DNA isolated from strain collection (B) strains, using between 10 and 15 clones for each strain. Apart from strain c/t13TU 10090, the degree of similarity between ITS sequences of individual clones for each strain was between 99.5 and 100%. Occasional single nucleotide polymorphisms (SNPs) were present in some ITS Types (see Figure 1 legend for definition). These are not considered to be sequencing artefacts since they were located repeatedly in the same positions in multiple replicate sequences of cloned ITS fragments generated here, and/or in retrieved deposited whole genome or ITS sequence data downloaded from GenBank. Furthermore, for the Acb strains whose ITS sequences were sequenced both directly and using colony PCR (see Materials and Methods), the similarities between the clone-based ITS consensus sequences and those derived from direct sequencing were always 100% [data not shown: accession numbers listed above].

The greatest number of Subtypes (defined in Figure 1 legend) were seen in *A. baumannii* (12 Subtypes), *A. nosoco*mialis (5 Subtypes) and *A. pittii* (8 Subtypes), (Figures 1 & 2).

### Features of *A. nosocomialis* ITS sequences

When ITS sequences from 12 *A. nosocomialis* collection A strains were downloaded from GenBank (see Table 1) and aligned, the sequences separated into one of two distinct Types (Figures 1 & 2): Type 607 (Group 1) and Type 615 (Group 2). Group 1 ITS sequences were all 607 bp and 99.7% similar to each other, while Group 2 sequences from the *A. nosocomialis* BCRC15417^T^ 615 bp ITS sequences generated here were 99.8% similar to each other (Table 1). The whole genome sequence of *A. nosocomialis* (strain AB22222) possessed three copies of Group 1 and one copy of Group 2 ITS sequences, while *A. nosocomialis* strains NCTC8102 (the same strain as BCRC15417^T^) and TG21145 contain only the Group 2 615 bp ITS variant. Consensus sequences were generated for ITS sequences of each of these Groups, and are referred to subsequently as *A. nosocomialis* (Group 1) and *A. nosocomialis* (Group 2) ITS respectively. The degree of similarity between these Group 1 and 2 ITS consensus sequences was only 96.4%.

During this study, no *A. nosocomialis* ITS sequences were sequenced by clone analysis, but ITS sequences homologous to Group 1 (Type 607, Subtype *A. nosocomialis*) were found in the sequenced collection (C) isolates, considered likely to be strains of *A. nosocomialis* from the 16S rRNA sequence data discussed above (strains 149, 153–6, 160, 163–8, 170–1, 176–8, 180–1, 248–9, 251–7). On the other hand, the Group 2 (Type 615, Subtype *A. nosocomialis*) ITS was seen only in strain 157 from collection (C) isolates (see Figure 1A and Figure 1 legend).

**Figure 3:**
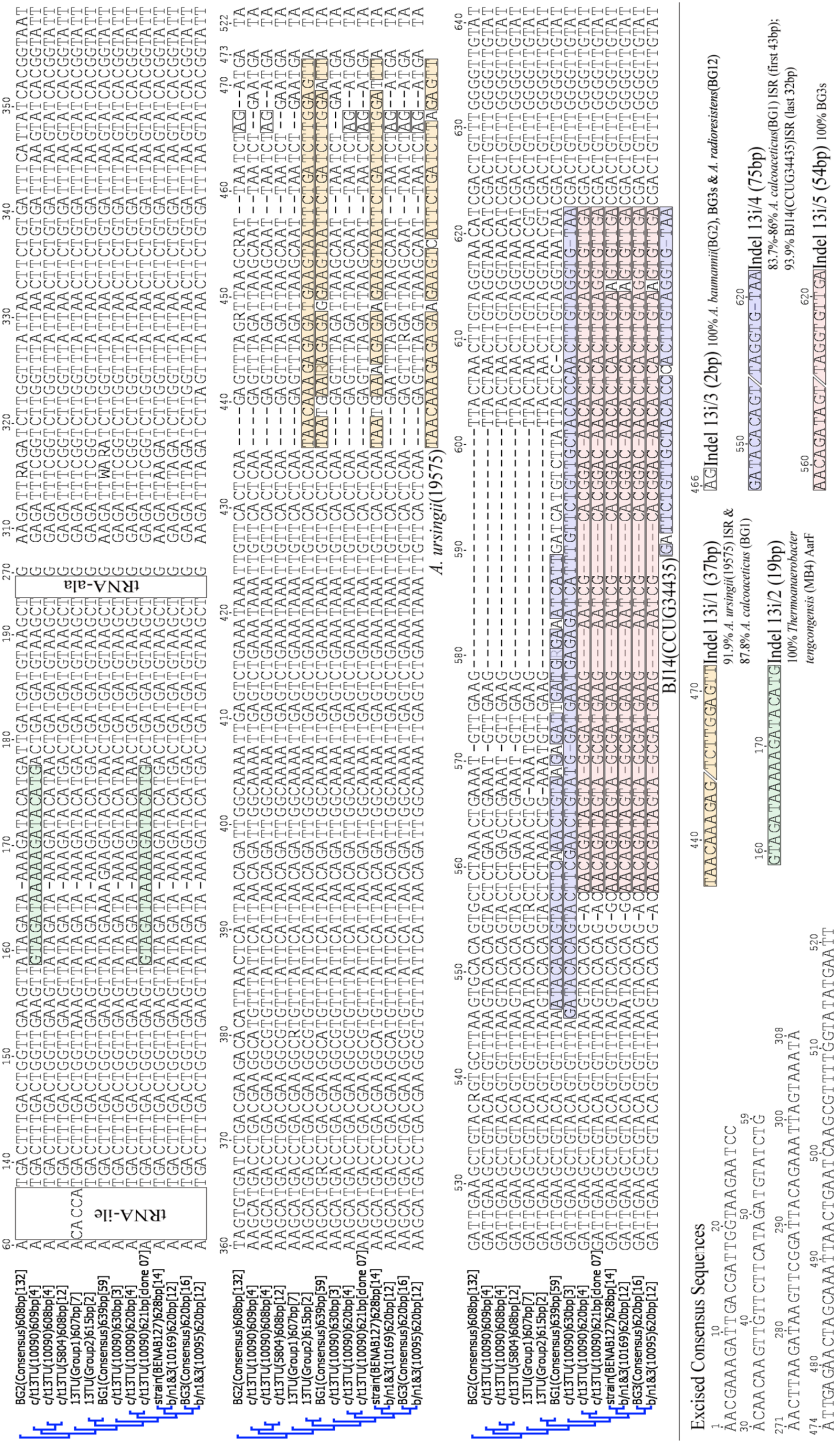
ITS consensus sequence alignments of Acb members including c/t13TU and *Acinetobacter* b/t 1 & 3 strains. For each indel, coloured bars are shown below and behind each sequence in the alignment, and closest Blast match listed below its name. Number of sequences used for each in [ ]. Sequences conserved for all species are shown as grey bars, and single nucleotide differences within as vertical lines coloured red (T), blue (C), green (A), black (G) and grey (N). Numbers at branch nodes are % consensus support using HKY genetic distance, neighbor joining tree and bootstrap resampling 100 replicates. *A. calcoaceticus* **(pink)**, *A. baumannii* **(yellow)**, *A. pittii* **(green)** and *A. nosocomialis* **(blue)** sequences are labelled BG1, BG2, BG3 and 13TU respectively.

Sequence analyses revealed a 37 bp region (TAA CAA AGA GAG ATG AAG TAA TTC TGA TCT TGG AGT T) distinguished the ITS sequences of ITS Group 1 (607 bp) from those of all Group 2 ITS (615 bp) *A. nosocomialis*. This 37 bp indel sequence is designated as indel 13i/1, and is present in the same location in the ITS of all Group 2 strains (Figure 3, position 436–472). Blast analysis showed its sequence is 91.9% similar to a region in the ITS sequence of *A. ursingii* LMG19575^T^, and 89.8% similar to a comparable region in the *A. calcoaceticus* ITS consensus sequence (Figures 2 & 3).

### Features of *Acinetobacter* c/t13TU strains 5804 and 10090 ITS sequences

No publicly available ITS sequences were available for strains c/t13TU 5804 and 10090. Data generated here showed that the ITS of strain c/t13TU 5804 were all 608 bp in length, and an ITS consensus sequence (from 11 clones, 99.8% similarity) was 98.8% similar to that of the *A. nosocomialis* Group1 (607 bp) ITS consensus sequence (Figure 2, Figure S1).

Alignment of ITS sequences of 16 strain c/t13TU 10090 clones revealed their lengths were more diverse than the single length 608 bp ITS detected in strain c/t13TU 5804. Thus, they fell into five distinct Types (Figure 1A, 609, 608, 620, 621, 630, denoted by an asterisk) of 608 bp (4 identical clones, and 99.1% similar to the 608 bp ITS sequence in strain 5804), 609 bp (4 clones, 99.9% similarity, and 99.5% similar to the 608 bp ITS in strain 5804), 620/1 bp (5 clones, 99.5% similarity) and 630 bp (3 identical clones), with an overall sequence similarity of only 95.8% (Figures 1 & 2, Figure S1).

A consensus sequence for each c/t13TU 10090 clone collection was then generated, but clearly this information can not be used directly for ITS-based identification, as the results obtained would depend upon which ITS Group consensus sequence was used. Thus, the c/t13TU 10090 608 bp, 609 bp, 620/1 bp and 630 bp ITS consensus sequences were 98.7%, 98.7%, 93.4% and 93.0% similar respectively to the *A. nosocomialis* Group 1 ITS consensus sequence (Figure S1), while the c/t13TU 10090 620 bp ITS consensus sequence was 99.4% similar to that of the *A. pittii* 620 bp ITS consensus sequence, as discussed below (Figure S1). Sequence data revealed that variability between the ITS sequences of the c/t13TU 10090 clone groups did not result from accumulated single nucleotide polymorphisms, but rather from the presence of four indels, designated here as indel 13i/2 to 13i/5 respectively (Figures 1, 2 & 3).

Blast analyses of these indels showed the closest matching sequences of indels 13i/3, 13i/4 and 13i/5) are found in the ITS sequences of other *Acinetobacter* species (Figures 1, 2 & 3). They also revealed that indel 13i/2 (19 bp: GTA GAT AAA AAG ATA CAT G ) is absent from the ITS sequences of all other *Acinetobacter* isolates so far examined (Maslunka, unpublished data). Instead, its sequence is identical to a region within the *AarF* gene of *Thermoanaerobacter tengcongensis* strain MB4 (position 1,187,443 to 1,187,462) (Figure S1), and it shares 18 of 19 nucleotides with a region in an undetermined gene in the genomes of *Bacillus cereus* strains Q1, AH187 and E33L (Q1 locus tag: BCQ_2928).

While indel 13i/3 (2 bp, underlined: ATT AAT CTA GAT GAA TTG) was too short to Blast, alignment data showed this same adjacent pair of nucleotides (A and G) and conserved flanking regions occur at the same locale in the *A. baumannii* ITS consensus sequence, and ITS sequences of all *A. pittii* strains so far sequenced. It is also present in the 620 bp ITS sequence of *Acinetobacter* b/n1&3 strains 10095 and 10169 (Figures 1, 2 & 3). The only available ITS sequence for the non-Acb member *A. radioresistens* BCRC15425^T^ also contains this indel sequence at the same locale (Figures 1, 2 & 3).

Indel 13i/4 is 75 bp long, and appears to have arisen from two recombination events. A Blast alignment of the first 43 nucleotides shows that all known ITS sequences of *A. calcoaceticus* strains have a homologous region at the same locale (83.7% to 86% similarity) (Figures 1, 2 & 3). The final 32 nucleotides are 93.9% similar to a region within *Acinetobacter* genomic species BJ14 CCUG34435^T^ ITS at a similar locale (Figures 2 & 3). Importantly, the first 43 nucleotide region of the *A. calcoaceticus* ITS sequence and the 32 nucleotide *Acinetobacter* BJ14 CCUG34435^T^ ITS region share a five nucleotide overlap (CAT TG) (Figure 3).

Indel 13i/5 is 54 bp long. It has a sequence identical to a comparable region in five of the seven *A. pittii* ITS sequences available in GenBank, and is 98.2% similar to those in the ITS sequence of *A. pittii* strains BCRC15420^T^ and DM21785–01. Its presence makes the *Acinetobacter* c/t13TU 10090 620 bp ITS sequence copies which carry indel 13i/5 essentially identical to the ITS sequences of all six *A. pittii* strains examined here (99.4% average similarity) (Figures 2 & 3), with only three nucleotides distinguishing the strain c/t13TU 10090 620 bp ITS sequence carrying indel 13i/5 from the *A. pittii* ITS consensus sequence (Figure 3, positions 177, 316, 318). The position 177 ‘A’ nucleotide is unique to the ITS sequence of strain c/t13TU 10090, and could conceivably be used to differentiate it from *A. pittii* by methods like High Resolution Melt-PCR (HRM-PCR) [23]. However, the latter nucleotide pair (AGA GAT TTC GG ) are of limited use for identification purposes as they are also found in the ITS sequences of all known strains of *A. nosocomialis*, as well as those of *Acinetobacter* c/t13TU 5804 (Figures 2 & 3).

Thus, there appear to be at least four different ITS sequence Types within the *rrn* operons of the genome of *Acinetobacter* c/t13TU strain 10090. The exact number remains to be determined by whole genome sequencing, although it can be concluded that the variations seen here in its ITS sequences share homology with those of *A. calcoaceticus* (Figure 2 red background: 608 and 609 bp) and *A. pittii* (see below) (Figure 2 green background: indel 13i/5, 621 bp).

### Features of *A. baumannii* ITS sequences

*A. baumannii* ITS sequences from 27 strains from GenBank (Table 1) and eleven whole genome sequences (Table 1) were aligned against those from 55 ITS clones from *A. baumannii* strains ATCC19606^T^, 97429, 97434, 16842 generated here. A single ITS consensus sequence was compiled. All 132 ITS sequences generated were 608 bp long and shared 99.7% homology. They represent 12 ITS Subtypes (See Figures 1 & 2, yellow background), with each differing by only a single nucleotide. The ITS sequences from *A. baumannii* are the shortest among the Acb members (608 bp), and do not contain the indels 13i/1, 13i/2, 13i/4 or 13i/5 (Figures 1 & 2).

Collection C clinical isolates 7–8, 150, 147–8 have the same ITS sequence Type and Subtype (608) as *A. baumannii.* Although those from isolates 8 and 150 differed from the other twelve *A. baumannii* subtypes by a single nucleotide (Figures 1 & 2), they would still fall into the *A. baumannii* Type (608) Group. Hence these data might suggest that collection C strains 7–8, 150 and 147–8 were probably identified correctly by 16S rRNA sequencing and pulse field electrophoresis as *A. baumannii*.

### Features of *A. pittii* ITS sequences

ITS sequences downloaded from GenBank from 10 *A. pittii* strains (single ITS sequence (Table 1), were aligned against those from 10 ITS clones generated here from *A. pittii* ATCC19004^T^. A single consensus sequence was generated as each ITS was 620 bp (Type 620) and they shared 99.7% sequence similarity. Four different sequence ITS Subtypes were detected within the genome of *A. pittii* strain D499, and a further eight were present in five other *A. pittii* strains (see Figure 1). Both *A. calcoaceticus* NC016603 and the Acb complex non-member species *A. oleivorans* DR1 share the same ITS sequence Type as *A. pittii* (620), but they differ in their sequence Subtypes by single nucleotide differences (Figures 1 & 2). Importantly indel 13i/5 sequence (Subtype 54/54) was present in all *A. pittii* ITS sequences examined here, including the complete *rrn* operon set from the whole genome of *A. pitti* D449 (Figure 1 & 2).

### Features of *A. calcoaceticus* ITS sequences

The *A. calcoaceticus* ITS sequences fell into two sequence lengths of 620 bp (Type 620) and 639 bp (Type 639) (Figure 1). Sequences from six *A. calcoaceticus* strains from GenBank (Table 1) were aligned against ITS sequences generated from clone sequencing by colony PCR of *A. calcoaceticus* strains ATCC23055^T^, 97366, 97420, and 97424. All were 639 bp and 99.5% similar to each other. ITS sequence Type (620) only included the two alleles extracted from the *rrn* loci of the whole genome sequence of *A. calcoaceticus* BG1 NC016603.

Again these ITS length differences arose from differences in their indel content. Indels 13i/1 Subtype BG1 and 13i/4 Subtype BG1 were present in all *A. calcoaceticus* Type (639) ITS sequences (Figures 1, 2 & 3). On the other hand, indel 13i/5 Subtype 54/54 occurred in all *A. calcoaceticus* Type (620) ITS sequences and those from collection (C) clinical isolates 151 and 159, but not in any of Type (639) ITS sequences (Figures 1 & 2). It may be that Type (620) and (639) ITS and their associated indels are mutually exclusive in *A. calcoaceticus*, a view substantiated by the observation that whole genome sequence data from strain *A. calcoaceticus* PHEA-2 with ITS Type (620) and associated indels, lacked ITS Type (639)

### Features of *Acinetobacter* b/n1&3 Strains 10095 and 10169 ITS sequences

No publicly available ITS sequences were available for *Acinetobacter* b/n1&3 strains 10095 and 10169. Therefore, all the ITS sequences reported here were obtained by clone sequencing from strains in collection (B).

The 620 bp ITS consensus sequences of *Acinetobacter* b/n1&3 10095 (from 12 clones, 99.9% similarity) and b/n1&3 10169 (from 13 clones, 99.9% similarity) were 99% similar to each other. Furthermore, ITS sequences from both strains possess the same indel 13i/5 Subtype (Red, 53/54) as the six *A. oleivorans* DR1 ITS alleles (each identical to each other). (Figures 1 & 2; Table 1). The 13i/5 sequences and hence ITS from the two b/n1&3 strains have a different indel 13i/5 Subtype (Red, 53/54) to that seen in the ITS of *A. pittii* ATCC19004^T^ and *A. calcoaceticus* NC016603. As described above, while *A. pittii* ATCC 19004^T^ and *A. calcoaceticus* NC016603 possess indel 13i/5 (Subtype 54/54) (Purple, 54/54: Figures 1 & 2), their ITSs differ from those of b/n1&3 strains (Subtype 53/54) by one and three nucleotides respectively (Figures 1 & 2). On this basis, both b/n1&3 strains emerge more closely related to *A. oleivorans* DR1 than to either *A. pittii* ATCC19004^T^ or *A. calcoaceticus* NC016603, even though *A. oleivorans* is not recognised currently as a member of the Acb complex.

### Features of other *Acinetobacter* ITS sequences from closely related strains

The *Acinetobacter* strain BENAB127 628 bp ITS consensus sequence derived from 13 identical clones was most closely related to that of *A. pittii* strain 19004^T^ (96.1% similar). It too contained indel 13i/5 SubType 53/54 (Figures 1 & 2) as do the ITS of *Acinetobacter* b/n1&3 strains 10095 and 10169 discussed above (96.3% and, 96.7% similarity respectively). Furthermore, the *Acinetobacter* BENAB127 ITS consensus sequence contains a variant of indel 13i/1 (Subtype BENAB) which has four single nucleotide substitutions compared to that of indel 13i/1 present in the *A. calcoaceticus* ITS consensus sequence (Figure 2, red background).

**Figure 4:**
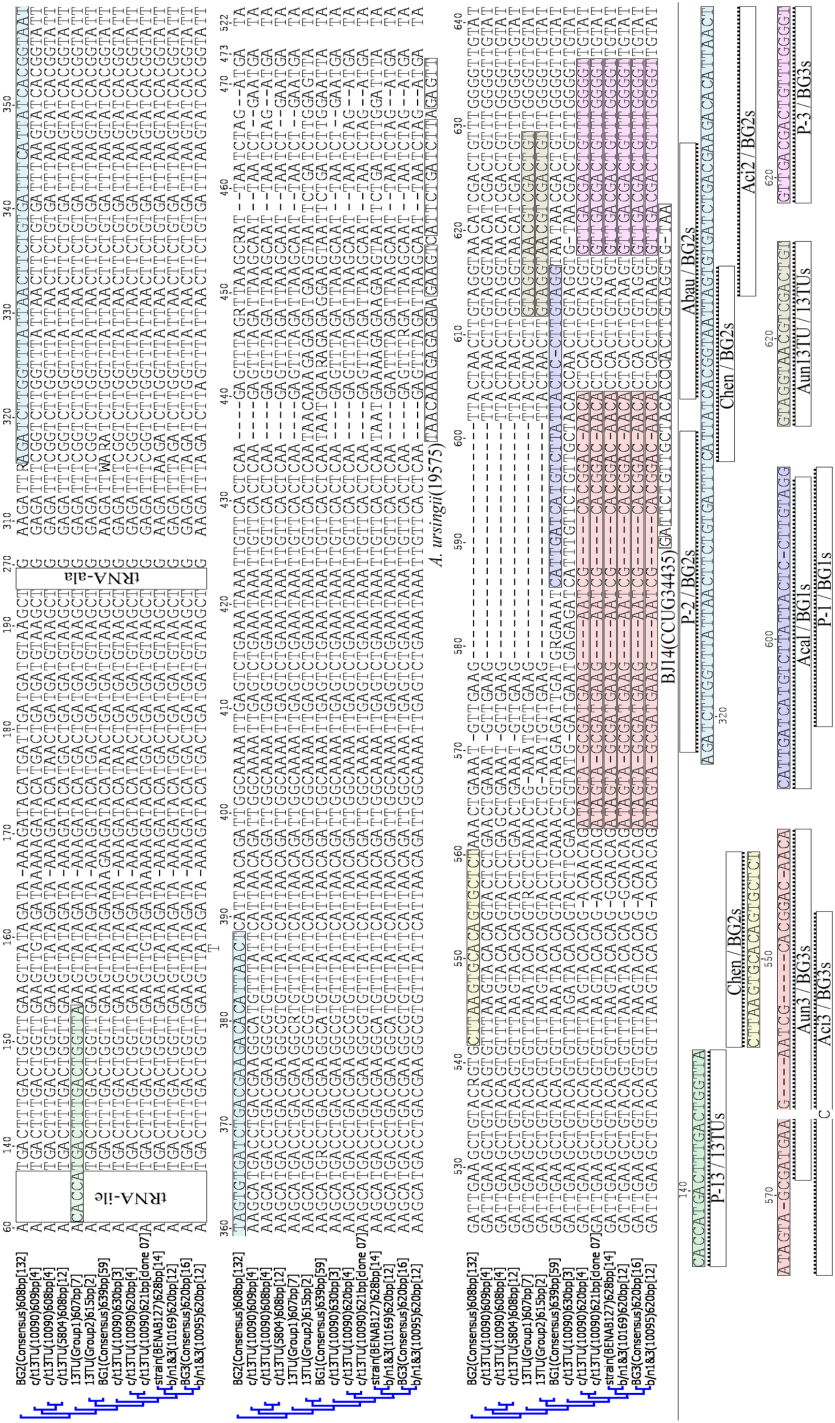
Alignment of ITS sequences of *Acb members* showing oligonucleotide probe target sites. These data show that existing oligonucleotide probes differentiate between the 620/1 bp ITS sequences of c/t13TU 10090, and those of genomic species *A. pittii* (BG3) and *Acinetobacter* b/n1&3 strains. Currently twelve probes have been designed to differentiate between Acb complex members: probes Acal, Abau and Aun3 [14]; probes Aci2 and Aci3 [16]; probes P-1, P-2, P-3, P-13 [17]; the two *A. baumannii*-targeted probes designed by Chen et al. [13] have not been designated names.

Analysis of the clone derived *Acinetobacter* BENAB127 ITS sequences would suggest the presence of only one ITS sequence Type (Type 628) although its *rrn* operon number is not known. Thus, it may be another closely related species to members of the *A. pittii* clade, but its ITS contains both indel 13i/1 (Subtype BENAB) (Figure 1C, light green) and indel 13i/5 Subtype 53/54 (Figure 1C, red), a combination not found in any other Acb strain ITS so far sequenced (Figures 2, 3 & 4).

ITS sequences from collection (C) strains 151, 159 and 169, which could not be placed into any prexisting Acb complex species by 16S rRNA sequence analyses, were most closely related to those from *A. pittii* (98.9%), c/t13TU (99.5–99.7%) and *A. oleivorans* (99.5%) and b/n 1&3 strains 10169 and 10095 (99.5%) respectively, all of which had ITS lengths of 620/622 bp. As mentioned above, the ITS of strains 151 and 159 both contain the indel 13i/5 subtype 54/54, while those of strains 159 and 169 contain indel 13i/3 and13i/5 subtype 53/54.

With collection (C) strains 175 and 250, their ITS sequences were most closely related to those of the *A. calcoaceticus* consensus sequence, but only with 96.1% similarity. The ITS sequences of both these strains lack the 13i/1 indel, but possess indel 13i/4 (subtype BG1) also present in *A. calcoaceticus.* Although, the sequence of ITS region 150–178 in strain 250 was not present in the *A. calcoaceticus* ITS consensus sequence, it was seen in the ITS of two putative *A. calcoaceticus* strains 1H9 and OCI1 [24]. On the other hand the ITS of strain 175 very closely resembled those of every other Acb member at this location. At positions 549–512, the ITS sequences of strains 175 and 250 were identical to that of the *A. calcoaceticus* strains, but different to those of all other Acb members in this region. Thus, these data reveal further ITS sequence variations, although it is not clear whether either of these strains represent a new *Acinetobacter* species, especially since the 16S rRNA sequence of strain 250 is identical to that of *A. calcoaceticus* ^T^ (data not presented here).

### Identification of Acb strains containing indel 13i/5 with the *A. pittii* targeted probe Aun3

**Figure 5:**
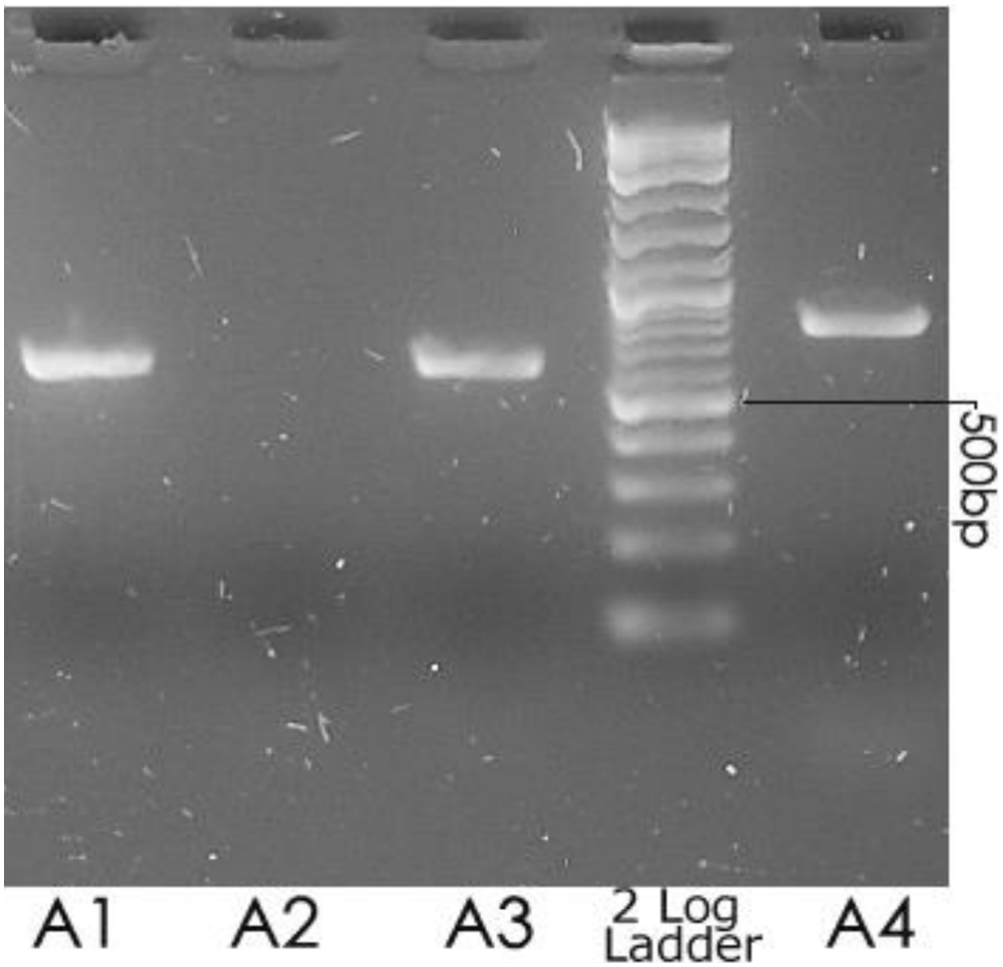
Electrophoretic separation of ITS amplicons from *A. pittii* ATCC19004^T^ (A1), c/t13TU 5804 (A2), and c/t13TU 10090 (A3). The amplification primers were 1512f and Aun3, designed to target only *A. pittii* strains [14]. A positive control was generated using primers 1512f and 6r to amplify the ITS sequences of strain c/t13TU 10090 (A4).

When the *A. pittii* ITS-targeted Aun3 probe [14] was used against *Acinetobacter* c/t13TU strain 10090, a PCR amplicon identical in size to that generated from *A. pittii* ATCC19004^T^ was obtained. Electrophoretic separation of the ITS amplicons on 2% agarose revealed that like *A. pittii* ATCC19004^T^, strain c/t13TU 10090 also produced a fragment of approximately 650 bp (Figure 5). The fragment size deduced from its ITS sequence alignment was 620 bp (Figure 3). No such fragment was generated from strain c/t13TU strain 5804, as its ITS sequence data would predict (Figure 4). Although not determined here, the same positive outcome would be expected for this probe when used against *Acinetobacter* b/n1&3 strains 10095 and 10169, and collection (C) strains 151, 159 and 169 too, since their ITS sequences also contain the Aun3 probe target site (Figure S1).

These results demonstrate elegantly what the sequence data in Figure 2 predict, namely that more than one species will be amplified by the indel 13i/5 targeted Aun3 oligonucleotide PCR primer, including *A. oleivorans*, *A. calcoaceticus* NC016603, *A. pittii*, *Acinetobacter* BENAB127, *Acinetobacter* c/t13TU 10090 and *Acinetobacter* b/n1&3 (10095 and 10169).

## Discussion

The feasibility of applying ITS sequences to identify reliably members of the *Acinetobacter* Acb complex has been addressed here. Earlier work had claimed that the length of the ITS region was an inherent characteristic for each Acb species, and hence could be used potentially as a primary tool for their individual identification [25]. This proposal seems to hold true for *A. baumannii,* where ITS copies of all strains appear to share the same length (608 bp), and where the only sequence differences are single nucleotide polymorphisms. Furthermore, both whole-ITS sequencing [9], [18] and ITS targeted oligonucleotide probes were considered as promising in being able to differentiate between members of the Acb and other *Acinetobacter* genomic species [13]–[17]. However, the potential complications caused by variable length ITS sequences in strains, as clearly demonstrated here, had not been addressed in these discussions.

Results presented here firmly establish for the first time that variable length ITS sequences in members of the *Acinetobacter* Acb complex result from the horizontal transfer of DNA fragments (i.e. indels), usually between the ITS sequences of species within this genus (Figure 3). In some instances, the presence of indels results in one or more ITS copy variants in strains of some Acb species being indistinguishable from those in others. Consequently, neither applying oligonucleotide probes nor whole-ITS sequencing, both still commonly used for identifying Acb members [26], [12], can be assumed always to distinguish between them.

Alignment analyses of ITS sequences from *A. calcoaceticus* (59 ITS sequences, 99.5% similarity) and *A. baumannii* (132 ITS sequences, 99.7% similarity) suggest the two *A. calcoaceticus*-targeted and six *A. baumannii*-targeted oligonucleotide probes available will differentiate them from other members of the Acb complex (Figure 4). In each case, multiple nucleotide variants distinguish the probe target sites from comparable regions in the ITS sequences of the other Acb species (Figure 4). While the high degree of similarity between each of the many *A. baumannii* ITS sequences analysed here may suggest indels are absent, this does not preclude them from acting as donors in horizontal gene transfer events.

The detection of indels like 13i/1 can be revealed only by comparing ITS sequences of the same strain/species. Thus, such analyses of ITS sequences of several *A. nosocomialis* strains revealed the 615 bp ITS sequences of strain BCRC15417^T^ were 8 bp longer than the 607 bp ITS sequences of the seven other *A. nosocomialis* (Group1) strains, because of the presence of the 37 bp indel 13i/1 (Figure 3). This finding addresses the concerns expressed by Zarilli et al. [18] regarding the distinctively different ITS length of most of their *A. nosocomialis* strains and the type strain BCRC15417^T^. It now appears that each of the less frequently seen 615 bp ITS copy sequences, regardless of the site of isolation of the host strains, contains this same indel 13i/1 located always in the same ITS position, which may result from a process of gene homogenization following its initial incorporation into a single ITS copy [27], [28], [29].

In contrast to the *A. nosocomialis* BCRC15417^T^ ITS sequences, where all copies contain the same indel 13i/1, the ITS sequences of *Acinetobacter* strain c/t13TU 10090 exhibit considerable intra-genomic variations, which arise from the variable insertion of four different indels (Figures 2 & 3). Relative to the shortest (608 bp) ITS in this strain, their presence increases the ITS length by 1 bp (13i/2), 0 bp (13i/3), 22 bp (13i/4) and 12 bp (13i/5) respectively (Figure 3). All these five indels appear to have arisen from horizontal gene transfer events between *Acinetobacter* species e.g. *A. calcoaceticus*, *A. baumannii*, *A. ursingii*, *A. pittii*, *A. nosocomialis*, *Acinetobacter* b/n1&3, *Acinetobacter* c/t13TU, *A. ursingii*, and *Acinetobacter* BJ14, or members of other genera (Figure 3). Establishing which species acts as donor/recipients is not possible, given the limited number of *Acinetobacter* ITS sequences available.

As a consequence of containing indels, some individual c/t13TU 10090 ITS sequences are less similar to each other than they are to the sequences of ITS copies in other *Acinetobacter* species. For example, while the *Acinetobacter* c/t13TU 10090 608 bp and 609 bp ITS sequences are 98.7% similar to those of *A. nosocomialis* Group 1, the presence of indel 13i/5 in the 620 bp ITS results in its sequence being 99.4% similar to the *A. pittii* 620 bp ITS consensus sequence (Figure 3). Thus, its presence makes it effectively impossible to distinguish between the 620 bp ITS sequences of *Acinetobacter* c/t13TU 10090 and *A. pittii* (Figures 4 & 5).

Based on phenotypic properties, ribotyping, plasmid profiling and DNA-DNA hybridization values, Gerner-Smidt & Tjernberg [5] suggested *Acinetobacter* c/t13TU strains 5804 and 10090 represented a new genomic species within the Acb complex. While *Acinetobacter* c/t13TU strain 5804 has only one ITS Type, 98.8% similar to that of the *A. nosocomialis* 13TU Group 1 (607) ITS consensus sequence, the variable presence of indels in ITS sequences of strain c/t13TU 10090 means its phylogenetic placement depends on whether 608 and 609 ITS Type copies are used (Figures. 2 & 3). In earlier attempts to clarify this, Nemec et al. [6] showed using *rpoB* gene sequences that both these strains clustered closely with *A. baumannii* and *A. nosocomialis*. Yet on the other hand, their *fusA, gltA* and *rplB* gene sequences suggested they were not closely related to either. Unfortunately, it may not be possible to use ITS sequencing to resolve this taxonomic question until whole genome sequence data from more c/t13TU strains strains of these become available [30]. The value of using ITS sequence data to distinguish between Acb species is further undermined when the sequences of *Acinetobacter* b/n1&3 strains 10095 and 10169 are examined. Both were also placed into a new genomic species in the Acb complex using criteria similar to those applied to the c/t13TU strains [5]. The suggestion is that the higher mutability (and hence variability) of ITS sequences may make them more suitable than 16S rRNA genes for distinguishing between closely related bacterial species or strains [31], [22], [32], [33]. However, no such variability was apparent in the 620 bp ITS sequences of b/n1&3 strains 10095 and 10169. All were essentially identical (99% and >99.2% similar respectively) to the *A. pittii* ATCC19004^T^ ITS sequence (Figure 3). Three possible conclusions arise from this: both are members of *A. pittii*, despite earlier data suggesting they represented distinct genomic species [5]; the ITS sequences of b/n1&3 strains 10095 and 10169 and those of the six *A. pittii* strains have not changed substantially since all eight strains last shared a common ancestor; their ITS sequences are so similar because of horizontal transfer of DNA fragments between them, possibly involving indel 13i/5 (Figure 3). It is not possible to determine which of these is the case until more *Acinetobacter* b/n1&3 strain ITS are sequenced.

Equally, the ITS sequences of *Acinetobacter* BENAB127 do not assist in resolving its possible relationship to existing Acb complex members. An alignment of its 628 bp ITS sequence against those of other Acb complex strains produced outcomes interpretable in one of two ways. One is that this strain is a member of *A. pittii*, and that each of its ITS sequences contains a variant of indel 13i/1 (Figures 1, 2 & 3). Alternatively, it might be argued equally that this strain is a member of *A. calcoaceticus*, and that each of its ITS sequences contains a variant of indel 13i/5 (Figures 1, 2 & 3). Similar problems arise in determining the taxonomic destiny of collection (C) strains 150, 159, 169, 175 and 250 (Figures 1, 2 & 3).

The presence of indels further complicates selection and application of the currently available ITS targeted probes to Acb members. If only a single ITS copy in a recipient strain possesses an indel which includes a region of DNA targeted by any probe, then its specificity is compromised. For example, *in* *silico* analysis of *Acinetobacter* c/t13TU 10090 620 bp ITS sequences reveals that the *A. pittii-*targeted probes Aci3 [16], P-3 [17] and Aun3 [14] would each return a positive result for *Acinetobacter* c/t13TU 10090, even though the 13i/5 indel is only carried in some, or possibly only one of its ITS copies. This prediction was confirmed experimentally, with strain c/t13TU 10090 returning a positive result for the Aun3 probe, while as expected, strain c/t13TU 5804 did not (Figures 4 & 5).

Unlike the *A. pittii*-targeted probes discussed above, neither of the *A. nosocomialis*-targeting Aun13TU [14] nor P-13 [17] probes appear to target ITS regions susceptible to recombination events (Figure 4). However, probe P-13 has been reported to cross hybridize with some *A. pittii* strains [17], which is not surprising given that ITS sequences of *A. nosocomialis* Group 1 strains differ from those of every other Acb strain by a single nucleotide only in the region targeted by this probe (Figure 4). Furthermore, since *A. nosocomialis* BCRC15417^T^ lacks the P-13 target site this probe will not target ITS sequences in all *A. nosocomialis* strains (Figure 4). In contrast, the Aun13TU probe [14] targets an ITS region occurring in all known *A. nosocomialis* strains (Figure 4). Its specificity depends entirely upon a single nucleotide difference in the middle of its ITS target region that distinguishes *A. nosocomialis* strains from both *A. baumannii* (42 strains) and c/t13TU strains 5804 and 10090 (Figure 4). The value of the Aun13TU probe is further undermined when a Blast search reveals the ITS sequences of both *A. radioresistens* BCRC15425^T^ (AY601839) and *Acinetobacter* genomic species BG6 BCRC15421 (AY601833) also contain its target site (Maslunka, unpublished).

One intriguing outcome of this work is that while individual ITS copies of many other *Acinetobacter* Acb species vary in their sequences from the presence of indels, those of the many different *A. baumannii* strains are all highly conserved. This is despite *A. baumannii* being naturally transformable [34], [35]. Diancourt et al. [36] also reported low levels of polymorphism in *A. baumannii,* which they suggested may indicate that *A. baumannii* either experienced a severe recent evolutionary bottleneck, or that the true diversity of this species is not represented by clinical isolates alone. While intra-genomic heterogeneity between bacterial ITS sequences resulting from homologous recombination is documented extensively [37], [38], [39], [27], [11], inter-genomic recombination events like those described here have never been reported previously for *Acinetobacter* ITS sequences. That the possibility of such events occurring has been overlooked in earlier studies is surprising, given the probable transformable nature of most *Acinetobacter* species [40], and that *Acinetobacter* ITS sequences contain, and are flanked by, highly conserved DNA regions (e.g. tRNA^ala^, tRNA^ile^, and the start/end of the 16S and 23S rRNA genes respectively). These data emphasize that a more cautious approach to using ITS sequences to identify individual *Acinetobacter* strains and species is needed, especially in those instances where variable length ITS sequences have been confirmed [41], [9]. An assessment of its usefulness as a target for species-specific probes/primers for other *Acinetobacter* species will only be possible when the whole genomes are sequenced in other strains [30] which contain variable length ITS copies.

## Supporting Information

Figure S1 **Alignments of c/t 13TU 10090 clone ITS sequences reveal presence of five indels.** The number of ITS sequences used in each consensus sequence is given in parentheses [ ].
(PDF)
