# Supplementary material for: Insertions or Deletions (Indels) in the rrn 16S-23S rRNA Gene Internal Transcribed Spacer Region (ITS) Compromise the Typing and Identification of Strains within the Acinetobacter calcoaceticus-baumannii (Acb) Complex and Closely Related Members
Source: PLoS One. 2014 Aug 20;9(8):e105390. doi: 10.1371/journal.pone.0105390 (PMC4139376; doi:10.1371/journal.pone.0105390)

Fig. S1. Alignment of c/t13TU 10090 clones ITS sequences reveals the presence of five indels. The number of ITS sequences used in each consensus sequence is given in parentheses [ ].

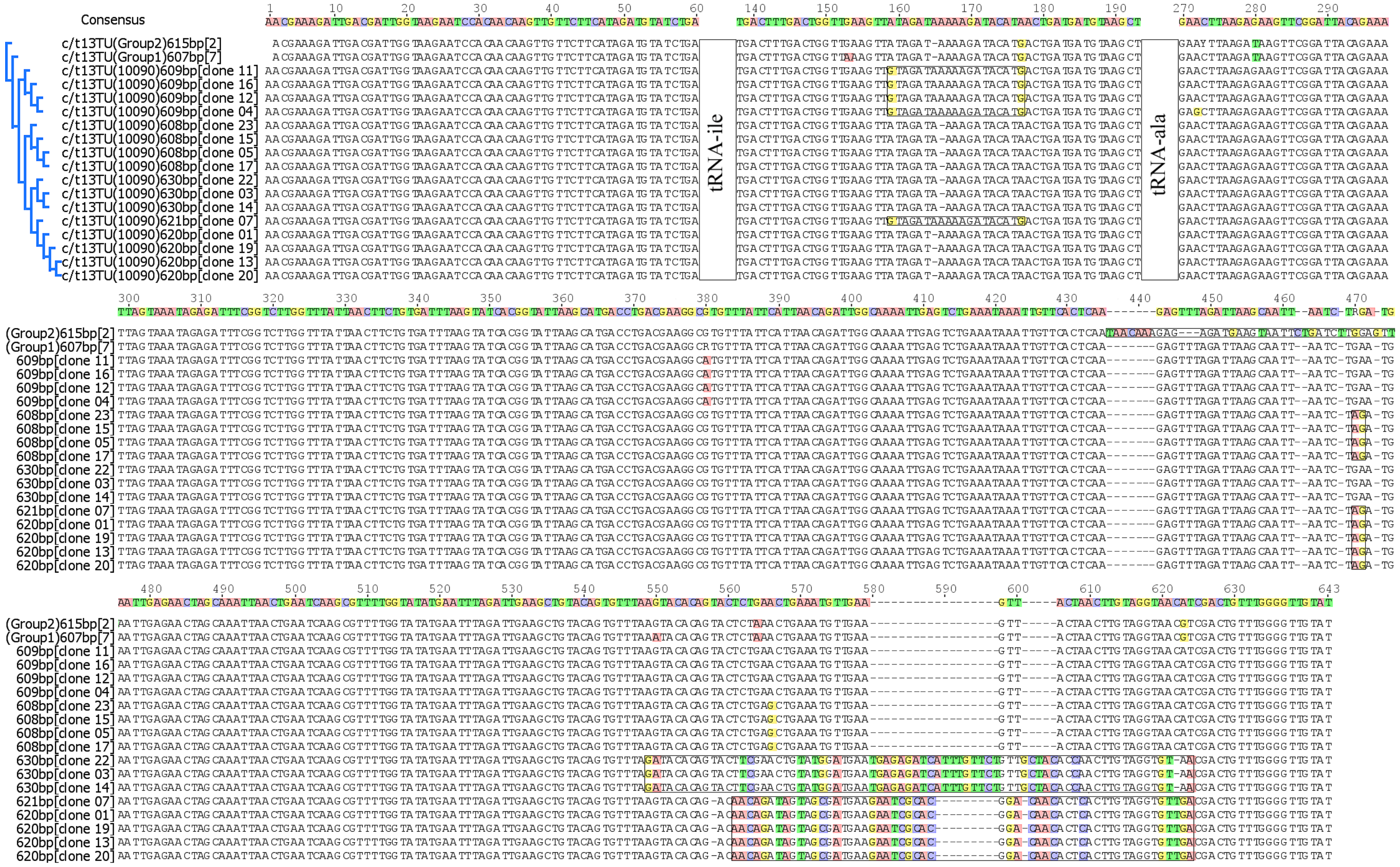

Supplement: Figure S1 — Alignments of c/t 13TU 10090 clone ITS sequences reveal presence of five indels. The number of ITS sequences used in each consensus sequence is given in parentheses [ ]. (PDF) [file pone.0105390.s001.pdf]
